# Stage-dependent niche segregation: insights from a multi-dimensional approach of two sympatric sibling seabirds

**DOI:** 10.1007/s00442-022-05181-0

**Published:** 2022-05-23

**Authors:** Aymeric Fromant, John P. Y. Arnould, Karine Delord, Grace J. Sutton, Alice Carravieri, Paco Bustamante, Colin M. Miskelly, Akiko Kato, Maud Brault-Favrou, Yves Cherel, Charles-André Bost

**Affiliations:** 1 School of Life and Environmental Sciences, Deakin University, 221 Burwood Hwy, Burwood, VIC 3125 Australia; 2 Centre d’Etudes Biologiques de Chizé (CEBC), UMR 7372 CNRS–La Rochelle Université, 79360 Villiers-en-Bois, France; 3 Littoral Environnement Et Sociétés (LIENSs), UMR 7266 CNRS–La Rochelle Université, 2 rue Olympe de Gouges, 17000 La Rochelle, France; 4 Institut Universitaire de France (IUF), 1 rue Descartes, 75005 Paris, France; 5 Museum of New Zealand Te Papa Tongarewa, PO Box 467, Wellington, 6140 New Zealand

**Keywords:** Niche partitioning, Foraging and diving behaviour, Trophic niche, Diving petrel, *Pelecanoides*

## Abstract

Niche theory predicts that to reduce competition for the same resource, sympatric ecologically similar species should exploit divergent niches and segregate in one or more dimensions. Seasonal variations in environmental conditions and energy requirements can influence the mechanisms and the degree of niche segregation. However, studies have overlooked the multi-dimensional aspect of niche segregation over the whole annual cycle, and key facets of species co-existence still remain ambiguous. The present study provides insights into the niche use and partitioning of two morphologically and ecologically similar seabirds, the common (CDP, *Pelecanoides urinatrix*) and the South Georgian diving petrel (SGDP, *Pelecanoides georgicus*). Using phenology, at-sea distribution, diving behavior and isotopic data (during the incubation, chick-rearing and non-breeding periods), we show that the degree of partitioning was highly stage-dependent. During the breeding season, the greater niche segregation during chick-rearing than incubation supported the hypothesis that resource partitioning increases during energetically demanding periods. During the post breeding period, while species-specific latitudinal differences were expected (species specific water mass preference), CDP and SGDP also migrated in divergent directions. This segregation in migration area may not be only a response to the selective pressure arising from competition avoidance between sympatric species, but instead, could reflect past evolutionary divergence. Such stage-dependent and context-dependent niche segregation demonstrates the importance of integrative approaches combining techniques from different fields, throughout the entire annual cycle, to better understand the co-existence of ecologically similar species. This is particularly relevant in order to fully understand the short and long-term effects of ongoing environmental changes on species distributions and communities.

This work demonstrates the need of integrative multi-dimensional approaches combining concepts and techniques from different fields to understand the mechanism and causal factors of niche segregation.

**Supplementary Information:**

The online version contains supplementary material available at 10.1007/s00442-022-05181-0.

## Introduction

The concept of niche is central in ecology, defined as a volume within a multi-dimensional niche space (Hutchinson 1957), and has found important applications in fundamental ecology, evolution, species management and conservation (Putman and Flueck 2011). Niche theory predicts that in order to limit the competition for the same resource, sympatric ecologically similar species should exploit divergent niches and segregate in one or more dimensions (MacArthur 1958). Niche segregation has been observed in a diverse range of taxa including plants (Monson et al. 1983), invertebrates (Finke and Snyder 2008) and vertebrates (Latham 1999), in both terrestrials and marine environments (Ainley et al. 2009). Investigating niche segregation is essential to gather knowledge about how and why species co-exist, especially for sibling species. In addition, it is also of particular relevance to evaluate the species' ability to adjust the characteristics of its niche over time and space. This step is an essential prerequisite for assessing a species’ capacity to buffer current and future environmental changes.

Seabirds are a particularly good model taxon to study niche segregation as they aggregate in large mixed-species assemblages in spatially constrained breeding and foraging habitats (Ainley et al. 2009). Although there is an ongoing interest in niche segregation in seabirds, the strong three-dimensional aspect of the marine environment challenges our understanding of the underlying mechanisms driving niche partitioning. In particular, segregation in seabirds can occur temporally (daily and seasonally; Granroth-Wilding and Phillips 2019), spatially (in both horizontal and vertical dimensions; Kokubun et al. 2016), and trophically (Cherel et al. 2005). However, very few studies have investigated the niche partitioning in more than two dimensions (Navarro et al. 2015), thereby complicating the possibility to distinguish the mechanisms leading to segregation.

High latitude seabirds typically experience varying influences of extrinsic and intrinsic factors throughout their annual cycle. Strong seasonal variations in oceanographic conditions and prey availability (extrinsic factors) can influence the patterns of niche partitioning, as trophic segregation might highlight competition for limited food resources, while a superabundance of prey enables overlapping niches (Barger and Kitaysky 2012). The degree of niche segregation might change according to the variation in energy requirements (intrinsic factors) related to the different constraints of each breeding stage and moult (Calado et al. 2018). During the breeding season, niche partitioning is likely to be at its maximum during the chick-rearing period, when offspring provisioning adds on to adult’s self-maintenance (Barger et al. 2016). Similarly, outside the breeding season, the high energetic demand of the moult may increase inter-species competition during this critical period (Dunn et al. 2019). However, most niche segregation studies have focused on one stage of the annual cycle at a time, mainly during the breeding season when seabirds are easily accessible. Therefore, key facets of species co-existence still remain ambiguous. Clearly, more attention is needed concerning the description of niche segregation throughout the entire annual cycle.

**Fig. 1:**
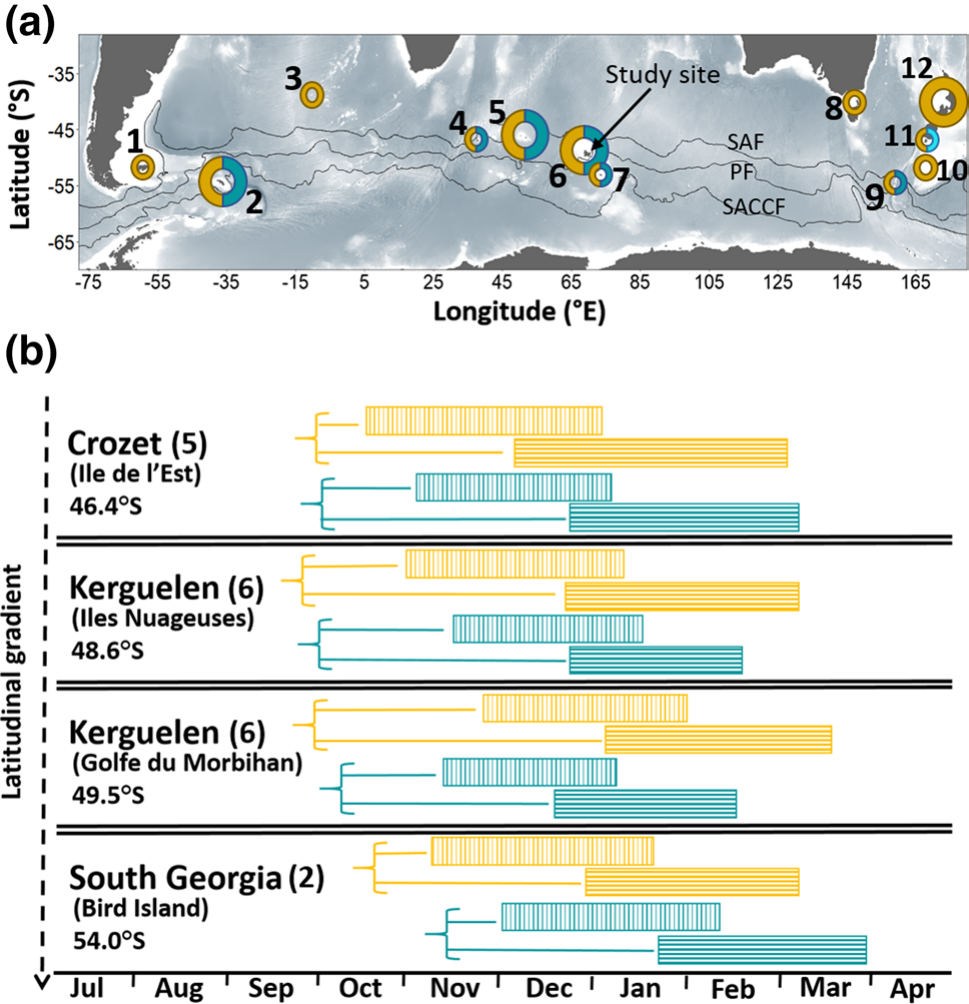
Upper panel (**a**): Distribution of common (CDP; yellow) and South Georgian (SGDP; blue) diving petrels. 1: Falklands/Malvinas Islands; 2: South Georgia; 3: Gough/Tristan da Cunha Islands; 4: Prince Edward Islands; 5: Crozet Islands; 6: Kerguelen Islands (study site); 7: Heard/McDonald Islands; 8: south-eastern Australia; 9: Macquarie Island; 10: Auckland/Campbell Islands; 11: Stewart Island islets; 12: New Zealand main islands. Distribution and population size data were acquired from Marchant and Higgins (1990). Larger circles show populations with more than 500 000 individuals of each species. Shared circles show sympatric populations of CDP and SGDP. Light blue for Stewart Island islets (11) corresponds to Whenua Hou diving petrel (*Pelecanoides whenuahouensis*, Fischer et al. 2018). The black lines represent the approximate location of the Subantarctic Front (SAF), Polar Front (PF) and Southern Antarctic Circumpolar Current Front (SACCF). Lower panel (**b**): Phenology of common (yellow) and South Georgian (blue) diving petrels breeding in sympatry. Blocks with vertical lines correspond to incubation, and horizontal lines show the chick-rearing period. Horizontal lines indicate the pre-breeding period (from when birds return to the colony to the start of the breeding period). Phenology data were adapted from Jouventin et al. (1985) for Crozet, Weimerskirch et al. (1989) and present study for Kerguelen, and Payne and Prince (1979) and Reid et al. (1997) for South Georgia. For Kerguelen, Ile Nuageuses are a group of offshore islands while Golfe du Morbihan is a semi-closed embayment. Analyses on phenology presented in the Results section include only data collected during the present study

The Southern Ocean hosts a wide range of sympatric seabirds with various physiological and ecological adaptations to the marine environment. However, conventional foraging studies (at-sea movements and diving behaviour) have focused mainly on large species mostly because of technological and practical reasons. Although these species provide valuable information on their environments, data collection has excluding a major part of the predator biomass that are small-sized seabird species. In addition, the primary investigation of niche segregation in such studies revolves around the flying characteristics of albatrosses (Phillips et al. 2004), or the diving capacities of penguins (Wilson 2010). Among seabirds of the Southern Ocean, diving petrels (*Pelecanoides* spp.) are unique in their diving (Navarro et al. 2014) and flying abilities (Fromant et al. 2021; Bost et al. 2022). In the five recognized species of diving petrels (Fischer et al. 2018; Marchant and Higgins 1990), the common diving petrel (CDP, *Pelecanoides urinatrix*) and the South Georgian diving petrel (SGDP, *Pelecanoides georgicus*) have a circumpolar distribution and breed sympatrically in several archipelagos of the Southern Ocean (Fig. 1; Marchant and Higgins 1990). Common and South Georgian diving petrels are pursuit divers (Ryan and Nel 1999), feeding mostly on macro-zooplankton (Ridoux 1994; Reid et al. 1997; Bocher et al. 2000; Fromant et al. 2020a). These two sibling species have been shown to locally segregate by foraging at different depths (Navarro et al. 2013; Bocher et al. 2000) and habitats (Navarro et al. 2015), or feeding on different prey (Ridoux 1994; Reid et al. 1997; Bocher et al. 2000). However, site-specific and stage-specific inconsistencies in their foraging ecology preclude a global picture of their segregation. In particular, the lack of investigations over the whole annual cycle, combined to the limited number of dimensions explored, complicate our ability to fully describe and understand the niche segregation of these two sympatric species.

We investigated the niche segregation between CDP and SGDP at Kerguelen Islands, by quantifying the spatial, temporal and trophic differences between these two morphologically and ecologically similar species throughout their whole annual cycle. Using an integrative approach combining phenology, at-sea movement, diving, accelerometer, and isotopic data, we addressed three main questions: (1) do CDP and SGDP differ in their timing of breeding, distribution, diving behaviour and isotopic niche?; (2) does the degree of niche segregation vary throughout their annual cycle?; and (3) are the processes leading to niche partitioning (niche specialization or competitive exclusion) similar during the pre-breeding, incubation, chick-rearing and post-breeding periods?

Based on previous trophic and isotopic analysis (Bocher et al. 2000) we predicted that niche segregation between the two species is mostly driven by (1) differences in diving behavior, spatial partitioning and diet during the breeding period and (2) spatial partitioning during the post-breeding period (similarly to what was observed among other small-sized procellariiform species; Quillfeldt et al. 2015). Since niche segregation can be more pronounced during energetically challenging periods (Barger et al. 2016), we also predicted stronger behavioural and/or trophic differences during the chick-rearing period, and during the first months of the post-breeding period, when adults renew their plumage.

## Methods

Fieldwork was conducted at Kerguelen Islands, Southern Indian Ocean. A total of 121 CDP and 105 SGDP were tracked across five consecutive annual cycles (see details of year- and stage-specific in Table S1). Although both species breed in sympatry on some islands of the archipelago, for logistical and practical reasons, the study colonies we used were located on two islands 6 km apart within the Golfe du Morbihan (semi-closed embayment): CDP at Ile Mayes (49°28’S, 69°57’E) and SGDP at Ile aux Cochons (49°47’S,70°05’E). Both species breed in burrows and the nest chamber was accessed by an artificial entrance covered with a removable stone lid. This access system reduced the disturbance of the natural tunnel and facilitated rapid access to the birds which were captured in the nest burrow for all procedures (Fromant et al. 2020b). The annual cycle was divided into four distinct periods: the incubation and chick-rearing periods during the breeding season, and the post-breeding migration (from departure to return to the colony) and pre-breeding period (from return to the colony to the start of the breeding season) during the non-breeding season.

To obtain an overview of the breeding phenology of both species, chicks were monitored and measured during the breeding season 2015–2016 (CDP = 25, SGDP = 27), and hatching dates were determined using the method described by Eizenberg et al. (2021). The wing length–age relationship was used as a proxy to back-calculate hatching date (see Supplementary materials for more details).

To evaluate the at-sea distribution and diving behaviour during both the incubation and chick-rearing periods, miniature GPS (2.0 g; nanoFix-GEO, Pathtrack Ltd., Otley, United Kingdom), time-depth recorder (TDR; 2.7 g; Cefas G5, Cefas Technology Ltd, Lowestoft, United Kingdom) and depth-accelerometer (4.0 g; AxiDepth, TechnoSmArt Ltd, Italy) data loggers were deployed using adhesive water proof tape (Tesa 4651, Beiersdorf AG, Germany) on two central tail feathers (for GPS and TDR) or on back feathers (for accelerometers). The GPS loggers were programmed to record locations at 10 min and 5 min interval during the incubation and chick-rearing periods, respectively. Both TDRs and depth-accelerometer data loggers were programmed to record pressure and hence dive depth (± 5 cm), and temperature (± 0.1 °C) every 1 s. In addition, accelerometers measured tri-axial body acceleration at 25 Hz. Because of the small size of the species (< 180 g), only one type of device was deployed on each individual at a time. The total mass of logger attachments was between 1.5 and 2.5% of body weight for CDP (120–180 g), and 2.0–2.9% for SGDP (110–150 g).

To determine the at-sea distribution of CDP and SGDP during the non-breeding period (post-breeding migration and pre-breeding period), adult birds were equipped with leg-mounted GLS (Migrate Technology, model C65, United Kingdom) (1.1 ± 0.1% of body mass). Breeding individuals were equipped at the end of the breeding season and were recaptured during the following breeding season.

Stable isotope ratios of carbon (δ^13^C) and nitrogen (δ^15^N) in whole blood and body feathers were used as proxies of the foraging habitat and diet/trophic level, respectively. Specifically, isotopic values of whole blood (hereafter blood) reflect dietary integration of approximately 2–4 weeks, while body feathers reflect dietary intake when they were synthesized (Cherel et al. 2000). Blood (0.2 mL) was collected from the brachial vein at recapture for stable isotope analysis and sexing. Sex was determined by DNA analysis (Laboratoire Analyses Biologiques, CEBC, France). Individuals were weighed (± 2 g; Pesola), and bill, tarsus (± 0.1 mm; Vernier calipers) and wing length (± 1 mm; ruler) were measured.

Processing of phenology data, spatial analyses (GPS and GLS data), diving analyses (dive depth recorder and accelerometer) and isotopic analyses are detailed in the supplementary materials. Statistical analyses were conducted within the R statistical environment (R Core Team 2020). Effects of species, stage, and year (fixed effects) on foraging and diving parameters were investigated by generating multiple Generalized Linear Mixed Models (GLMMs) using the package *glmmADMB* (Bolker et al. 2012). Individual was added as a random effect. To investigate factors influencing diving behaviour (dive depth, dive duration and mean VeDBA per dive; VeDBA = Vectorial Dynamic Body Acceleration, see Supplementary text for more information), Generalized Additive Mixed Models (GAMMs) were fitted using the *mgcv* package (Wood 2018). Models were ranked based on their Akaike’s Information Criterion (AIC) and were checked to ensure normality and homoscedasticity of residuals (Zuur et al. 2010) before further statistical analyses. *Post-hoc* tests were conducted using non-parametric statistics (Kruskal–Wallis and Mann–Whitney *U* tests) when parametric test assumptions of normality were not met. To investigate at-sea spatial segregation, the percentage overlap in foraging distribution was estimated using Bhattacharyya’s Affinity (BA) index (Fieberg and Kochanny 2005) using the *adehabitatHR* R package (Calenge 2006). BA index (0 signifying no overlap in UDs, and 1 = complete overlap) is a statistical measure for the degree of similarity amongst UDs, and the amount of space-use shared among species. Inter-species variations in phenology (laying, hatching and fledging dates) were tested using *t*tests (parametric), or Mann–Whitney *U* tests (non-parametric) depending on the data distributions. The isotopic niche position and width were compared between species and breeding stages using the ellipse area-based metrics of the *SIBER* package (Jackson et al. 2011).

All the morphological measurements, trip parameters and stable isotopes results, were compared between the sexes for both species (Table S2). Because of the limited inter-sex differences for both CDP and SGDP, data were pooled in all subsequent statistical analyses. Similarly, because of the small inter-annual variations in foraging behavior and stable isotope values, data were pooled by species and stage.

## Results

### Morphological differences

**Fig. 2:**
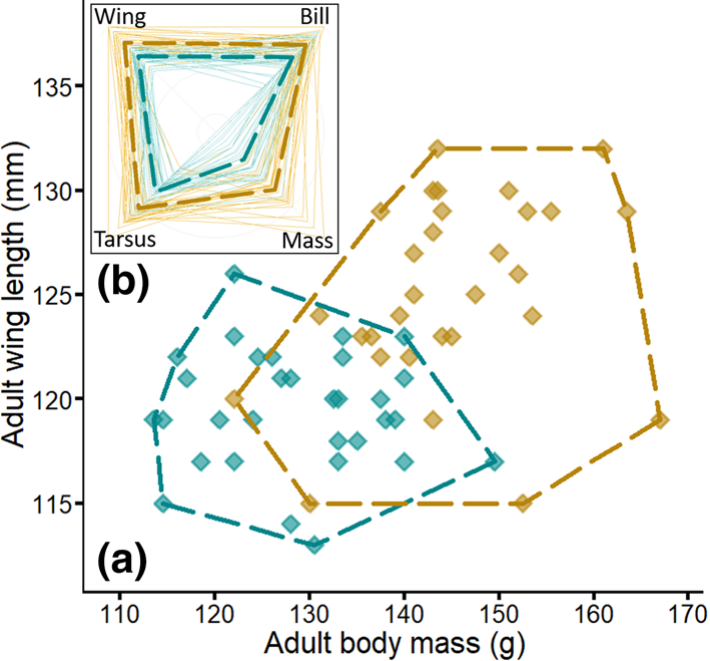
Main panel (**a**): morphological differences between adult common (yellow) and South Georgian (blue) diving petrels breeding at Kerguelen Islands. The dashed lines indicate the morphological range of each species. Top left panel (**b**): radial chart indicating the intra- and inter-species morphological variations. These are relative values estimated as the proportion of the maximum individual values for both species combined (Value/Maximum Value (CDP : SGDP)). Each faint line corresponds to one individual, and the bold dashed lines correspond to the mean value for each species

Morphological differences between CDP and SGDP were investigated using measurements of body mass, wing length, tarsus length, and bill length of adult breeding individuals. Although all the measurements overlapped between the two species (Fig. 2), CDP had significantly larger body mass, and longer wing, tarsus and bill lengths (Table S3). The difference between the two species was emphasized by CDP being proportionately heavier than SGDP (Fig. 2), resulting in a higher wing loading (assuming proportionate wing shape; ratio body mass/wing length, CDP = 1.16 ± 0.08; SGDP = 1.08 ± 0.08; *t* tests: *t*_60.001_ = 4.146, *P* < 0.001).

### Phenology

The average laying date of CDP (28-Nov ± 10; ranging from 15-Nov to 18-Dec) was estimated to be 18 days later than SGDP (10-Nov ± 10; ranging from 27-Oct to 12-Dec; *t* tests: *t*_47.238_ = − 6.291, *P* < 0.001). Similarly, owing to the longer incubation and chick-rearing duration period for CDP, the mean hatching date of CDP (22-Jan ± 10) was estimated to occur on average 26 days later than SGDP (27-Dec ± 10; *t* tests: *t*_47.238_ = − 9.082, *P* < 0.001), and the fledging date was 32 days later for CDP (16-Mar ± 10) than SGDP (13-Feb ± 10; *t* tests: *t*_47.238_ = − 11.176, *P* < 0.001). The incubation period of CDP overlapped during 29 days of the SGDP incubation period (62% overlap), and also 26 days with the SGDP chick-rearing period (55% overlap) (Fig. 1).

### Spatial segregation: at-sea distribution and diving behavior

**Fig. 3:**
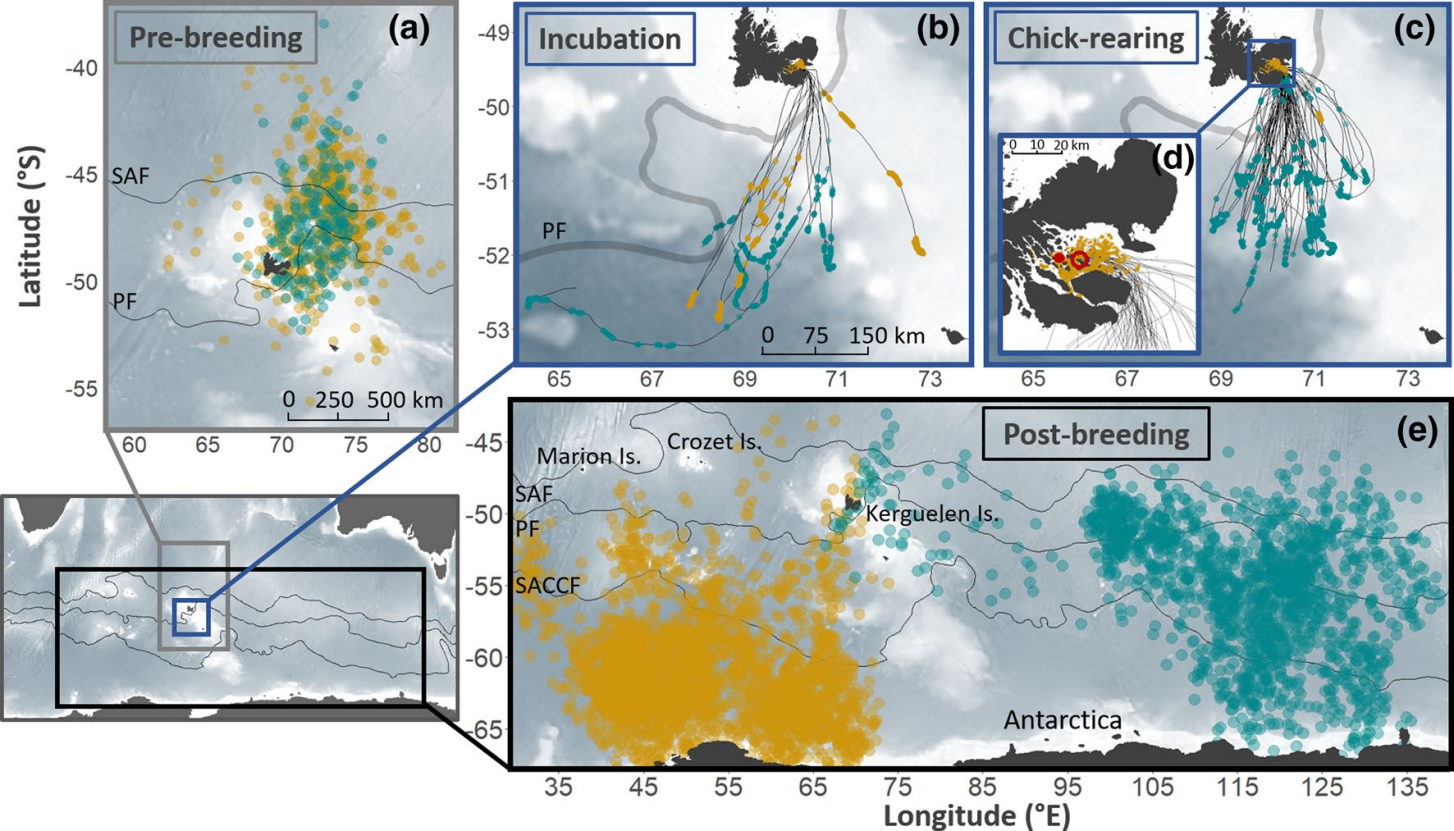
At-sea distribution of common (yellow) and South Georgian (blue) diving petrels from Kerguelen Islands. Data in pre-breeding (**a**) and post-breeding (**e**) were collected using GLS (2 locations per day). Tracks in incubation (**b**) and chick-rearing (**c** and **d**) were collected using GPS, where dots indicate positions with speed < 9.1 km·h^−1^ (proxy of foraging locations; see Methods for more details). In panel **d**, the full red circle indicates the location of Ile Mayes where CDP were studied, and the open red circle the location of Ile aux Cochons where SGDP were studied

During the pre-breeding period, both CDP and SGDP travelled at-sea north-east of the Kerguelen Plateau within 1000 km of their colony locations (Fig. 3a). During this period, the at-sea distribution of CDP and SGDP completely overlapped (BA indices for 50% UDs = 0.94).

**Fig. 4:**
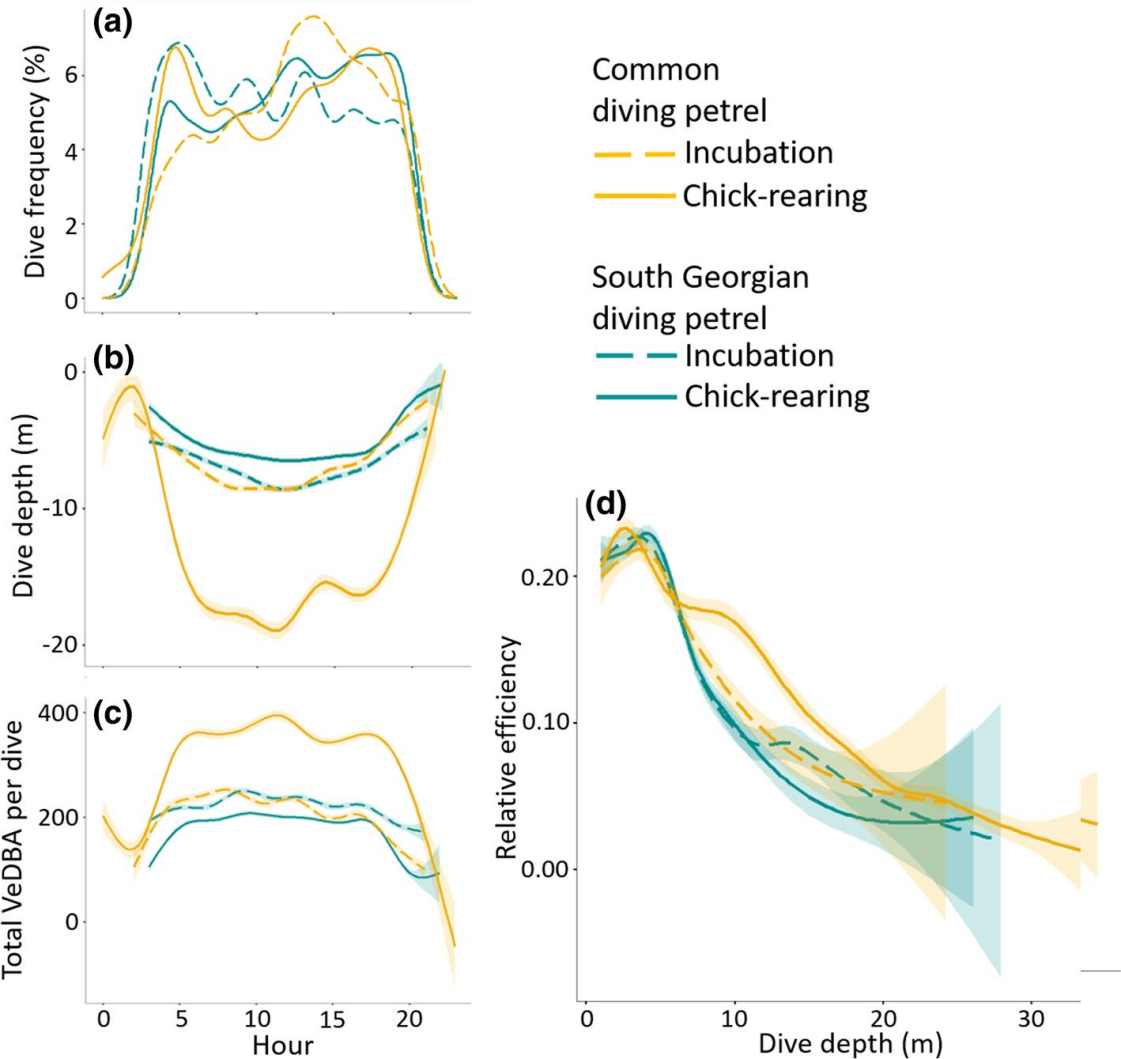
Hourly variation for dive frequency (**a**), dive depth (**b**) and total VeDBA (Vectorial Dynamic Body Acceleration) (**c**) and relative diving efficiency (**d**) (Wilson 2010) of common and South Georgian diving petrels during both incubation and chick-rearing periods predicted by generalized additive mixed models. The efficiency is calculated by dividing the duration of the bottom phase by the total time spent during one dive cycle (dive duration + post-dive duration) for that particular depth

During the incubation period, both CDP and SGDP travelled 200–400 km south of Kerguelen, along the shelf-slope of the Kerguelen Plateau (Fig. 3b), overlapping in their foraging distribution (BA indices for 50% UDs = 0.62). Although one CDP individual undertook a short foraging trip within the Golfe du Morbihan, there was no significant difference for offshore trips between both species, neither in duration (CDP = 45 ± 1 h; SGDP = 45 ± 24 h; Mann–Whitney *U* test: *U* = 3, *P* = 0.700), nor in distance travelled (CDP = 777 ± 154 km; SGDP = 653 ± 98 km; Mann–Whitney *U* test: *U* = 7, *P* = 0.400) or maximum distance from the colony (CDP = 331 ± 71 km; SGDP = 322 ± 108 km; Mann–Whitney *U* test: *U* = 14, *P* = 0.762). Similarly, CDP and SGDP exhibited comparable dive characteristics during the incubation period (Table 1; Fig. 4). Both species were diving to similar depths (Mann–Whitney *U* test: *U* = 14, *P* = 0.808), and for similar durations (Mann–Whitney *U* test: *U* = 24, *P* = 0.214). Nonetheless, CDP and SGDP differed in their mean VeDBA values. While diving at a similar depth, SGDP exhibited higher mean VeBDA values than CDP (Fig. S1), for both dive duration (*F*_6.020_ = 178.60, *P* < 0.01) and depth (*F*_6.215_ = 165.03, *P* < 0.01), indicating they were more active underwater than CDP.

**Table 1:** Overall tracking data and whole blood stable isotope values (mean ± SD) of common and South-Georgian diving petrels during the incubation and chick-rearing periods at Kerguelen Islands

	Common diving petrels		South-Georgian diving petrels	
	Incubation	Chick-rearing	Incubation	Chick-rearing
GPS data (*N* individuals; *n* trips)	*N* = 6; *n* = 6	*N* = 31; *n* = 39	*N* = 6; *n* = 6	*N* = 37; *n* = 46
Trip duration (h)	40 ± 10^a^	19 ± 1^b^	45 ± 24^a,c^	28 ± 10^c^
Total distance travelled (km)	506 ± 388^a^	84 ± 23^b^	653 ± 98^a^	535 ± 115^a^
Maximum distance from colony (km)	227 ± 171^a,b^	19 ± 10^c^	322 ± 108^a^	208 ± 68^b^
Dive data (*N* individuals; *n* trips)	*N* = 7; *n* = 7	*N* = 12; *n* = 21	*N* = 4; *n* = 4	*N* = 11; *n* = 13
Dive depth (m)	6.5 ± 0.5^a^	15.2 ± 3.2^b^	6.6 ± 0.8^a^	6.1 ± 2.6^a^
Dive duration (s)	28 ± 3^a^	44 ± 6^b^	25 ± 3^ac^	23 ± 5^c^
Time activity budget (*N* individuals; *n* trips)	*N* = 7; *n* = 7	*N* = 10; *n* = 19	*N* = 4; *n* = 4	*N* = 6; *n* = 8
Flying (%)	53.4 ± 14.0^ab^	50.9 ± 12.9^b^	33.0 ± 4.8^a^	49.2 ± 19.0^ab^
Resting (%)	27.5 ± 13.5^ab^	25.6 ± 12.9^b^	49.9 ± 4.2^a^	33.4 ± 18.7^ab^
Diving (%)	19.1 ± 5.1^a,b^	23.5 ± 4.7^b^	17.1 ± 3.2^a^	17.4 ± 3.3^a^
Stable isotopes (*N* individuals)	*N* = 46	*N* = 50	*N* = 22	*N* = 51
Whole blood δ^13^C (%)	− 21.3 ± 2.1^a^	− 18.4 ± 1.2^b^	− 23.3 ± 0.3^c^	− 22.7 ± 0.4^d^
Whole blood δ^15^N (%)	9.2 ± 1.2^a^	11.0 ± 1.0^b^	9.0 ± 0.3^a^	8.4 ± 0.4^c^

For each parameter, values not sharing the same superscript letter (a, b or c) are significantly different (Mann–Whitney *U* test: *P* < 0.05)

During the chick-rearing period, CDP and SGDP strongly segregated in their at-sea distribution, dive depth, and dive duration (Table 1; Fig. 3c, d). While SGDP continued to forage at a distance, along the shelf-slope of the Kerguelen Plateau, CDP foraged inshore, within the Golfe du Morbihan. This switch in foraging habitat by CDP resulted in a decrease in the prospecting distance. This was associated with an increased diving effort for CDP, with birds diving significantly deeper (Mann–Whitney *U* test: *U* = 2, *P* < 0.001) and longer (Mann–Whitney *U* test: *U* = 1, *P* < 0.001) than SGDP at that time (Table 1; Fig. S2). The relationship between dive bottom duration, post-dive duration and dive depth indicated divergent relative dive efficiencies (Fig. 4d), with CDP being more efficient divers than SGDP with increasing depths.

During the post-breeding period, CDP and SGDP differed markedly in their at-sea distributions. Directly after the breeding season (1–5 days after the last burrow attendance), both species migrated in divergent directions (2000–5000 km apart; Fig. 3e). The maximum migration range was significantly larger for SGDP (Mann–Whitney *U* test: *U* = 8, *P* = 0.016; Table S4), as well as the total distance travelled (Mann–Whitney *U* test: *U* = 8, *P* = 0.006) and the total duration of migration (Mann–Whitney *U* test: *U* = 8, *P* = 0.002).

### Isotopic niche

**Fig. 5:**
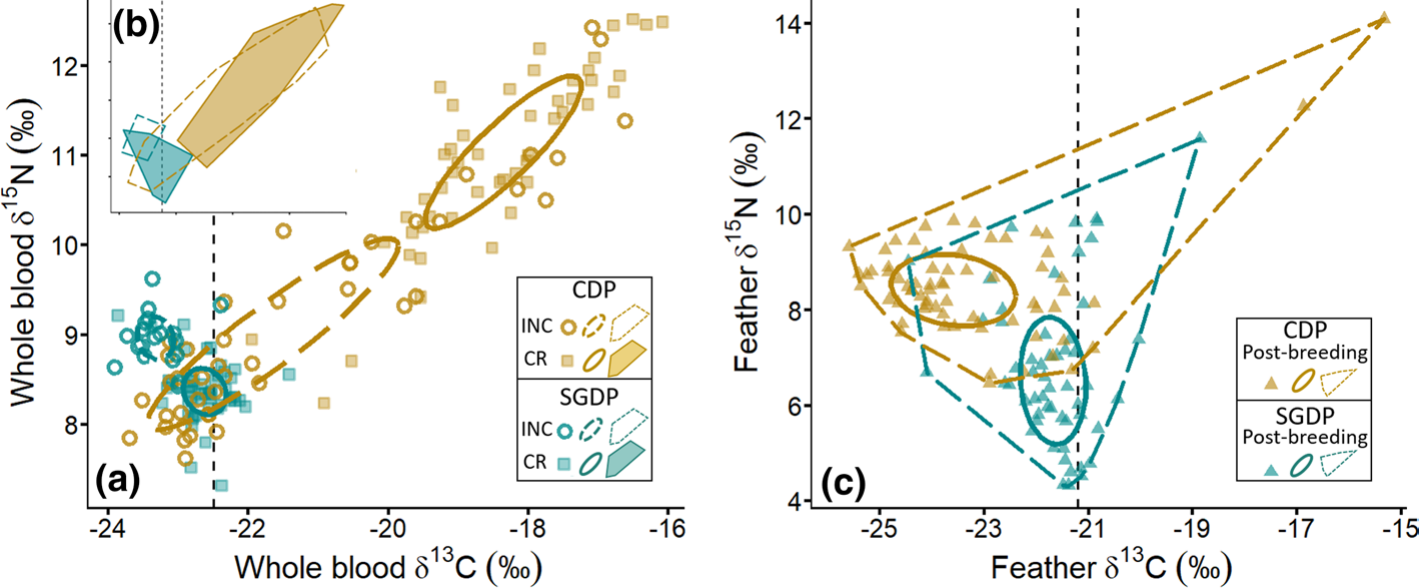
Values of δ^13^C and δ^15^N values in blood (**a** and **b**) and feathers (**c**) of common (yellow) and South-Georgian (blue) diving petrels from Kerguelen Islands. The main left panel (**a**), with standard ellipses corrected for sample size (SEAc), corresponds to incubation (INC) and chick-rearing (CR) periods, and the top left panel (**b**) indicates the total convex hull area (total amount of niche space occupied)**.** The right panel (**c**) corresponds to the post-breeding period, with the standard ellipses corrected for sample size and the total convex hull area. The black vertical dashed line corresponds to the value for the Polar Front

During incubation, despite total convex hull areas partially overlapping (Fig. 5a, b), stable isotope values in blood were significantly different between species for *δ*^13^C (*t* tests: *t*_50.095_ = 6.391, *P* < 0.001), but not for *δ*^15^N (*t* tests: *t*_54.974_ = 1.371, *P* = 0.175). While inter-individual variation for SGDP was low (Table S5; Fig. 5a, b), isotopic values of incubating CDP stretched out into the following two groups: a low-value group (*δ*^13^C < − 21.5%, and *δ*^15^N < 9.5%), and a high-value group (− 21.5 < *δ*^13^C < − 17%, and 9.5 < *δ*^15^N < 12.5%). The first group, comprising the majority of CDP samples, showed similar *δ*^13^C values between the two species, but lower *δ*^15^N values than SGDP (Fig. 5a).

Total convex hull areas did not overlap during chick-rearing, both species fully segregating in their isotopic signatures during this stage (Fig. 5b; for *δ*^13^C, *t*_58.359_ = 22.803, *P* < 0.001; *δ*^15^N, *t*_60.663_ = 17.648, *P* < 0.001). All SGDP exhibited low *δ*^13^C and *δ*^15^N values close to those of CDP during incubation (Fig. S3; Table S5; all *δ*^13^C < − 21.4% and *δ*^15^N < 9.3%), while CDP showed almost exclusively higher values with *δ*^13^C > − 20.0% and *δ*^15^N > 10%.

During the non-breeding period (moulting period; Fig. 5c), CDP exhibited significantly lower feather *δ*^13^C values (*t* tests: *t*_234.57_ = − 9.943, *P* < 0.001) and higher *δ*^15^N values than SGDP (*t* tests: *t*_166.07_ = 10.206, *P* < 0.001). SGDP had a larger range of *δ*^15^N values than CDP, including a group of low values (< 5.0%). Conversely, both species had few outliers that were characterized by both high *δ*^13^C (> − 20%) and *δ*^15^N (> 11%) values (Fig. 5c).

## Discussion

This study provides unique insights into the niche segregation of two congeneric species throughout their whole annual cycle, by combining at-sea movement, diving, accelerometer, and isotopic datasets. The degree of partitioning was highly stage-dependent, emphasized by the shift from limited segregation during the incubation period to complete niche segregation during the chick-rearing period (Table 2). Such seasonal variation supports the hypothesis that resource partitioning between sympatric similar species increases during energetically demanding periods. The variation between breeding stages was likely related to differences in the processes involved in niche segregation, such as competitive exclusion or niche specialization. In post-breeding, the complete separated migration paths and overwintering grounds of CDP and SGDP may involve processes other than inter-species niche segregation, such as past evolutionary divergence.

**Table 2:** Summary of spatial (at-sea distribution and dive depth) and trophic segregation between common (CDP) and South-Georgian (SGDP) diving petrels from Kerguelen Islands during the whole annual cycle

	Degree of segregation	Comment
Pre-breeding		
Distribution	−	Overlapping (both species off-shore)
Dive depth		No data
δ^13^C (%)		No data
δ^15^N (%)		No data
Incubation		
Distribution	−	Overlapping (both species mostly foraging off-shore)
Dive depth	−	CDP = SGDP
δ^13^C (%)	+	Large distribution of values for CDP
δ^15^N (%)	+	For similar δ^13^C, CDP < SGDP
Chick-rearing		
Distribution	+ + +	Full spatial segregation (CDP foraging inshore)
Dive depth	+ + +	CDP > > SGDP
δ^13^C (%)	+ + +	CDP > > SGDP
δ^15^N (%)	+ + +	CDP > > SGDP
Post-breeding		
Distribution	+ + +	Full segregation (latitudinal and longitudinal)
Dive depth		No data
δ^13^C (%)	+ +	CDP < SGDP
δ^15^N (%)	+ +	CDP > SGDP

The degree of segregation is symbolized as a gradient from no segregation (−) to strong segregation (+ + +)

### Phenology: influence of oceanographic conditions

In the present study, SGDP started breeding 2–3 weeks earlier than CDP, which was in accordance with historical data from the study site (Weimerskirch et al. 1989). However, this marginal allochrony is inconsistent with the general pattern observed elsewhere in the Southern Ocean (e.g. South-Georgia, Crozet and Kerguelen offshore islands), where CDP typically begin breeding slightly earlier than SGDP (Fig. 1). Timing of breeding is a species/population specific life history trait (Perrins 1970), expected to be synchronized with optimal environmental conditions according to the species ecology. The similar phenology of SGDP populations throughout the species distribution highlights a preference for foraging in offshore waters, where the timing of maximum productivity is constant at a large spatial scale (Labat et al. 2005). Conversely, the substantial variation in the phenology of CDP at both large and local scales (Weimerskirch et al. 1989; Fromant et al. 2020c) may be driven by a stronger influence of local inshore conditions (Weimerskirch et al. 1989).

Interspecific differences in timing of breeding can also be interpreted as a mechanism to reduce competition between ecological similar species (Granroth-Wilding and Phillips 2019). Nevertheless, the relatively long incubation duration of CDP tends to extend the period during which both species share the same foraging area (i.e. during both the incubation and the early chick-rearing periods of SGDP). This, in addition to the important inconsistencies between breeding sites, downplays the importance of such slight allochrony as a mechanism to reduce competition.

### Incomplete segregation in the early breeding period: competitive exclusion theory

Both CDP and SGDP shared similar pelagic foraging areas during the pre-breeding and incubation periods. For both species, the similarity was emphasized by a clear shift in foraging area from the north-east part of the Kerguelen Plateau in pre-breeding, to the south during the incubation period. Such habitat switching between pre-breeding and incubation is common within seabirds (Cherel et al. 2014; Quillfeldt et al. 2020) and is likely to be related to the limited range that a diving petrel can reach between two incubation shifts (1–3 days; Fromant et al. 2021). As central place foragers, breeding seabirds must find a trade-off between performing short enough foraging trips and accessing productive areas. While the north-eastern sector of the Kerguelen Plateau is highly productive (Blain et al. 2007), its distant location (500–800 km from the study colonies) may force CDP and SGDP to exploit a closer foraging area, matching the requirements of undertaking short incubation shifts.

The exploitation of waters along the south-western shelf-slopes by both species during incubation was characterized by similar trip parameters (trip duration, distance travelled and dive depths). However, and despite large overlap in their isotopic niche, stable isotope analyses revealed subtle trophic differences. For similar values of *δ*^13^C (proxy indicating similar water mass), SGDP exhibited slightly higher *δ*^15^N values than CDP suggesting that both species may partially differ in their targeted prey. Although both diving petrel species are known to feed on pelagic euphausiid and copepods species (Bocher et al. 2000), the knowledge of their diet during the incubation period is still limited.

During the incubation period, in addition to the difference in blood *δ*^15^N values, SGDP exhibited higher diving effort (higher mean VeDBA) than CDP, despite similar dive characteristics (dive depth and duration). The exploitative competition theory (Wootton 1994) predicts that the larger species (CDP) forages more efficiently, thus outcompeting and excluding the smaller one (SGDP). The larger species occupies the niche where intake rates are highest while minimizing diving effort, whereas the smaller species is constrained to increase its diving effort to catch any other available high-quality food. In the scenario where both species target and forage on similar prey patches, the higher effort observed in SGDP imply that they must swim harder to access remaining prey. To supplement their intake under competition, SGDP may need to forage on larger prey that are potentially harder to catch (Reid et al. 1997), resulting in the observed higher blood *δ*^15^N values and diving effort when compared to CDP. In addition, when the two species fully segregate during the chick-rearing period (see next section), SGDP occupy the niche left vacant by CDP. In particular, this is illustrated by the shift of SGDP isotopic niche towards the niche previously occupied by CDP, supporting the exploitative competition hypothesis. Yet, while body size difference appears to be the main factor driving competitive exclusion, and has been largely documented in various cases in both terrestrial and marine environments (Wearmouth and Sims 2008), our understanding of such predator–prey interactions will remain unclear without direct observation of foraging behaviour in the field.

### Complete niche segregation in chick-rearing: niche specialization theory

During the chick-rearing period, CDP and SGDP fully differed in their at-sea distribution, diving behavior and isotopic niche. This substantial change in the degree of segregation between the two species was driven by a drastic shift in CDP foraging ecology. While SGDP foraged in similar offshore areas and depths during both the incubation and chick-rearing periods, CDP foraging habitat during chick-rearing was restricted to the coastal area (Golfe du Morbihan), switching from open ocean to a semi-closed embayment. This resulted in a substantial decrease in trip duration and distance travelled than during incubation, and when compared to both the incubation and chick-rearing periods of SGDP. This profound shift in at-sea distribution of CDP during the chick-rearing period coincided with substantial modifications in their diving behaviour (increased depth, duration and mean VeDBA per dive).

The change in foraging niche occupied by CDP during the incubation and chick-rearing periods is further supported by the substantial shift in isotopic niche. This complete spatial and isotopic niche segregation has been previously illustrated by stomach content analyses, showing that CDP rely mostly on the swarming amphipod *Themisto gaudichaudii* during this period (Bocher et al. 2000). In the Golfe du Morbihan, this crustacean displays a strong seasonal variation with a peak of abundance in summer (Labat et al. 2005), precisely matching the chick-rearing period of CDP. As income breeders (Chastel et al. 1995), diving petrels are expected to match the energy-demanding chick-rearing period with a peak of resource availability (Perrins 1970). Thus, it is likely that the observed switch in CDP foraging habitat between incubation and chick-rearing is triggered by the summer high density of *T. gaudichaudii* in the Golfe du Morbihan (Bocher et al. 2001).

In addition, the overall pattern of isotopic values shifting from offshore to inshore environments between incubation and chick-rearing masks the fact that some CDP individuals already started feeding in the Golfe du Morbihan while still incubating. This suggests that CDP switched foraging behaviour as soon as *T. gaudichaudii* became available within the gulf. Such results may provide key information to understand the process of niche segregation between CDP and SGDP. Indeed, surprisingly, SGDP did not appear to take advantage of this reliable and locally superabundant prey during neither the incubation period nor the energetically demanding chick-rearing period.

Spatial and trophic segregations are considered to result from competitive exclusion (the bigger species accessing the best resource), or niche specialization (induced by morphological and/or physiological differences) (Phillips et al. 2004). Although, both processes may be applicable in the present case, the total absence *T. gaudichaudii* from the SGDP trophic niche strongly suggests partitioning arising from physical capabilities and diving performance. Indeed, for a breath-hold diving species, maximum dive depth and duration generally increase with body mass (Schreer and Kovacs 1997; Halsey et al. 2006), while prey capture is proportional to the time that an individual allocates to the bottom phase of a dive (Wilson 2010). Following the model of depth/time relative efficiency developed by Wilson (2010), CDP appears to be the most efficient of the two species at depths in excess of 10 m.

The relative absence of surface feeders in the Golfe du Morbihan (Bocher et al. 2001; Cherel et al. 2014), in addition to the depths exploited by CDP and coastal penguin species feeding on *T. gaudichaudii* (Bocher et al. 2000, 2001), confirms that this abundant prey is mainly restricted to depths deeper than 10 m. Therefore, the relatively lower efficiency of SGDP at deeper depths, associated with their smaller body size, may restrict their access to *T. gaudichaudii* in the Golfe du Morbihan. Although SGDP are able to dive as deep as 20 m, when compared to CDP for similar dive depth and duration, the higher mean VeDBA observed for SGDP suggests these individuals have a lower diving capacity.

In addition, the lower wing loading of SGDP may also reflect their adaptation to flying over longer distances than CDP (Thaxter et al. 2010). Notable differences in diving performances and energetic expenditure of the SGDP appear to be key factors explaining the use of distant areas to target more accessible prey species at shallower depth. For SGDP, the energetic cost of repeated deep and long dives may exceed the cost of undertaking longer trips but foraging on more accessible prey in conditions of limited exploitative competition. Therefore, the complete niche segregation observed between CDP and SGDP during the chick-rearing period may result from niche specialization and not direct competition.

In the extensive literature exploring niche partitioning between similar species or sex, niche specialization commonly originates from body size differences and divergent relative efficiencies to exploit the environment (Wearmouth and Sims 2008). For example, the between-sex difference in wing loading for albatrosses may advantage females in lower wind conditions (Phillips et al. 2011), which ultimately appears to induce latitudinal habitat specialization (Weimerskirch et al. 1997; Phillips et al. 2004). Similarly, niche segregation in alcids during the chick-rearing period appears to be caused by differential flying and/or diving capabilities (Thaxter et al. 2010).

### Post-breeding migration: historical distribution and congeneric segregation

During the inter-breeding period, CDP and SGDP headed in divergent directions and different latitudes. The stable isotopic signatures in body feathers supported the idea of latitudinal segregation, with lower *δ*^13^C values for CDP indicating a moulting area farther south than for SGDP (Jaeger et al. 2010). Adult diving petrels migrate to wintering areas directly after the end of the breeding season (Rayner et al. 2017; Fromant et al. 2020c) and renew their plumage during the first months of this period (Fromant et al. 2020c). Because moult is an energetically/nutritional demanding process, seabirds are likely to renew their plumage where the surrounding waters are productive (Cherel et al. 2016), which may incite ecologically similar species to migrate to different areas. Previous studies on winter distribution of small petrels and prions showed clear inter-species latitudinal segregation, which was explained by differences in preferred water masses (Quillfeldt et al. 2015).

Interestingly, the recent studies investigating the post-breeding distribution of three different diving petrel species (Navarro et al. 2015; Rayner et al. 2017; Fromant et al. 2020c; Fischer et al. 2021; present study) all revealed that diving petrels migrate to well-defined population/species-specific areas. This contrasts with the highly dispersive behaviour generally observed with other small-sized procellariiform species (Quillfeldt et al. 2015; Navarro et al. 2015). In particular, the ecological theory of segregation predicts that individuals should disperse when they are no longer tied to their breeding grounds. Therefore, the observed segregation in migration area may not be a response to the selective pressure arising from present competition avoidance between sympatric species, but instead, could reflect past evolutionary divergence (Peck-Richardson et al. 2018). Divergent but consistent species-based and population-based cultural patterns may suggest that each species/population is responding to different life history traits. The evolution of wintering ecological optimum for each species/population may, therefore, involve historical distribution shift of water masses but also the sequence of colonization (s) and speciation within diving petrels.

In addition, by heading south-east, Kerguelen SGDP may as well segregate from large populations of conspecifics breeding on Crozet Islands. Two species can indeed segregate in other dimensions than space when their distribution overlaps, while two synchronous populations of the same species must segregate spatially in order to avoid competition for the same resource. Thus, perceived inter-breeding segregation between sympatric sibling species could rather result from intra-species (populations) competition avoidance.

## Conclusion

Overall, the present study demonstrates the importance of integrating approaches from different fields (foraging and trophic ecology, ecophysiology, phenology and morphometry) to describe the co-existence of ecologically similar species. The degree of partitioning and the mechanisms involved were highly stage-dependent, allowing a better understanding in the coexistence of large populations of two sibling seabird species. Although any study of niche segregation is only a snapshot of a continuous process, such results point to multiple, non-exclusive causal factors of niche segregation. The evolution of species optimum through competition may have lead SGDP to exploit a niche where CDP are absent or rarely present (as seen during the incubation period). Alternatively, both species have evolved separately, and developed different capacities/preferences related to their optimal environment (such as the segregation observed during the chick-rearing and post-breeding period). In the context of climate change, the fragile equilibrium between species living in sympatry is likely to be modified. Investigating the ecology and niche segregation of ubiquitous species experiencing rapid environmental modifications is, therefore, fundamental to fully understand the short and long-term effects of climate change.

## Supplementary Information

Below is the link to the electronic supplementary material.

Supplementary file1 (DOCX 1116 KB)

## Data Availability

Our data are available within the Dryad Digital Repository: https://datadryad.org/stash/share/RLL_JzEDtHvSrHBxFlp8vLP5LAn0KULj-JOpeARG8tA.
