# Supplementary material for: Stage-dependent niche segregation: insights from a multi-dimensional approach of two sympatric sibling seabirds
Source: Oecologia. 2022 May 23;199(3):537–48. doi: 10.1007/s00442-022-05181-0 (PMC9309125; doi:10.1007/s00442-022-05181-0)
Supplement: Supplementary file 1 — Supplementary file1 (DOCX 1116 KB) [file 442_2022_5181_MOESM1_ESM.docx]

**Supplementary materials**

**Stage-dependent niche segregation: insights from a multi-dimensional approach of two sympatric sibling seabirds**

Aymeric Fromant, John P.Y. Arnould, Karine Delord, Grace J. Sutton, Alice Carravieri, Paco Bustamante, Colin M Miskelly, Akiko Kato, Maud Brault-Favrou, Yves Cherel, Charles-André Bost

**Supplementary text:**

**Data processing**

*Phenology*

Access constraints to the breeding colonies of CDP and SGDP, and the breeding period distributed over several months (large intra-species heterogeneity of laying dates; Marchant and Higgins 1990), precluded the collection of accurate data of laying, hatching, and fledging dates. Therefore, to obtain an overview of the breeding phenology of both species, chicks were monitored and measured during the breeding season 2015-2016 (CDP = 25, SGDP = 27), and hatching dates were determined using the method described by Eizenberg et al. (2021) where wing length–age relationship was used as a proxy to back-calculate hatching dates. Approximations of laying and fledging dates were then estimated using the duration of the incubation (CDP = 55 d; SGDP = 47 d) and chick-rearing (CDP = 54 d; SGDP = 48 d) periods (Payne and Prince 1979; Jouventin et al. 1985). For each chick, the hatching date was averaged using 2-4 different measurements over a three-week period.

*GPS data*

All GPS data were processed within the R statistical environment (R Core Team 2020). Prior to behavioral modeling, land-based points were removed and a speed filter with a threshold at 20 m·s^-1^ was applied to remove erroneous locations (Spear and Ainley 1997). Because of poor satellite reception during intense diving activity, linear interpolation was necessary to correct for unequal sampling frequencies between foraging and commuting. Foraging trips were defined as the time spent at sea between the departure from and the return to the burrow. For each complete trip, the following basic parameters were calculated: trip duration, total horizontal distance travelled, and maximum distance from the colony. Incomplete trips were only used to estimate maximum distance from the colony. Because diving petrels forage by diving from the sea surface, two behavioral states were identified: flying, and foraging/resting (foraging hereafter). To enable the comparison between CDP and SGDP, the discrimination between both states was determined using the conservative instantaneous speed threshold method (Petalas et al. 2021):

Speed threshold = 2*(Drift Speed * Average Flight Speed) / (Drift Speed + Average Flight Speed).

The average speed threshold of 9.1 km·h^-^1 was obtained assuming a drift speed of 5 km·h^-^1 (Petalas et al. 2021), and an average flight speed of 50 km·h^-^1 for both species. Average flight speed was estimated from the analysis of flying bouts using the raw data. Regular distance between location points over large distances (35 to 225 km) were used to select continuous flying bouts, and speed was averaged for each species (CDP = 49 ± 13 km·h^-1^; SGDP = 50 ± 11 km·h^-1^). Values lower to this threshold were assigned as foraging, and the remaining positions (instantaneous speed > 9.1 km·h^-1^) were defined as flying.

*GLS data*

Processing and calculations of GLS data were conducted using the *GeoLight* package in the R statistical environment (Lisovski et al. 2012, R Core Team 2020). The device records the maximum light intensity for each 5 min interval, and the determination of morning and evening twilights enables longitude (timing of local midday and midnight) and latitude (duration of day and night) to be estimated, providing two positions per day with an average accuracy of 186 ± 114 km (mean ± SD; Phillips et al. 2004). Filtered locations were used to generate kernel utilization distribution (UD) estimates using the same method as Fromant et al. (2020). The 50% (core foraging area) and 95% (home range) kernel UD contours were obtained. Spatial analyses were performed using the *adehabitatHR* R package (Calenge 2006).

*Diving activity and acceleration*

Dive data obtained from depth recorders and accelerometers were corrected for depth drift and processed using *diveMove* package (Luque and Fried, 2011). For each dive, the following dive parameters were calculated: time at the beginning and end of a dive, dive duration, dive depth, duration of descent, bottom time, ascent duration, and post-dive interval. The species relative dive efficiency was estimated by dividing the bottom duration during dives by the total time spent during one dive cycle for that particular cycle (Wilson 2010).

Accelerometer data were filtered to separate dynamic acceleration attributed to animal movement from static acceleration using a 1 s running mean. The Vectorial Dynamic Body Acceleration (VeDBA) was calculated using the following equation:

$$VeDBA = \surd(X_{dyn}^{2}+Y_{dyn}^{2}+Z_{dyn}^{2})$$

where X, Y and Z are the dynamic acceleration (dyn) of horizontal (surge), vertical (heave) and lateral (sway) movements, respectively. Mean and Total VeDBA per dive were used as proxies for diving effort (Qasem et al. 2012). Behavioral modes were identified from the combined accelerometer and depth data through *k*-means clustering analysis in the *Ethographer* package in IgorPro (Wavemetrics Inc., Portland OR, USA, version 6.3.7.2) (Sakamoto et al. 2009). Briefly, this method is a type of unsupervised clustering analysis that groups similar kinds of signals into clusters and was performed on the x-axis due to its greater variation. Three categories (surface resting, flying, and diving) were identified from the accelerometers, and were further classified using depth data to discriminate between the high amplitude observed in both flying and diving behavioural modes. The duration of each of the behaviour categories were determined for each individual and summed to obtain daily activity budgets.

*Stable isotopes*

Like for most small-sized procellariiforms, the moult of body feathers for diving petrels is a protracted process happening mostly after the end of the breeding season (Carravieri et al. 2014; Fromant et al. 2020). The Southern Ocean is marked by a strong latitudinal isotopic gradient, with δ^13^C and δ^15^N decreasing with increasing latitudes (Jaeger et al. 2010). In addition, at the local scale, coastal waters are generally characterized by high δ^13^C and δ^15^N baselines that propagate throughout the food web up to top predators (Cherel et al. 2014), thus allowing discrimination of coastal and pelagic foraging grounds of seabirds. Isotopic analyses were carried out on blood and body feathers to investigate the isotopic niche during the breeding (incubation and chick-rearing) and post-breeding periods, respectively. For the post-breeding period, four body feathers were collected from the middle and lower back of each individual tracked with a GLS. Additional samples were collected on other breeding individuals in order to increase the sample size. Blood was freeze-dried, ground to powder and homogenized, and sub-samples were weighed (0.4 mg) with a microbalance. To remove surface dirt, feathers were washed in a chloroform-methanol solution and oven dried for 24h as described by Carravieri et al. (2013). For each individual, feathers were homogenized by cutting them with scissors into small fragments. The relative abundance of carbon and nitrogen isotopes was determined with a continuous-flow mass spectrometer (Thermo Scientific Delta V Advantage) coupled to an elemental analyser (Thermo Scientific Flash EA 1112). Isotopic results are presented in the δ notation relative to Vienna PeeDee Belemnite and atmospheric nitrogen (N_2_) for δ^13^C and δ^15^N, respectively. Replicate measurements of internal laboratory standards (acetanilide) indicate meas­urement errors <0.10 ‰ for both δ^13^C and δ^15^N values. The C:N mass ratios of the samples were calculated as the ratio between the mass percentages of carbon and nitrogen. The consistently low C:N values (<4.0, Post et al. 2007) verified that the low lipid content of blood did not necessitate lipid extraction (Cherel et al. 2005).

**Literature cited**

# Calenge C (2006) The package adehabitat for the R software: a tool for the analysis of space and habitat use by animals. Ecol Modell 197:516–519.

# Carravieri A, Bustamante P, Churlaud C, Cherel Y (2013) Penguins as bioindicators of mercury contamination in the Southern Ocean: birds from the Kerguelen Islands as a case study. Sci Total Environ 454:141-148.

# Carravieri A, Bustamante P, Churlaud C, Fromant A, Cherel Y (2014) Moulting patterns drive within-individual variations of stable isotopes and mercury in seabird body feathers: implications for monitoring of the marine environment. Mar Biol 161:963–68.

# Cherel Y, Hobson KA, Weimerskirch H (2005) Using stable isotopes to study resource acquisition and allocation in procellariform seabirds. Oecologia 145:533–540.

# Cherel Y, Connan M, Jaeger A, Richard P (2014) Seabird year-round and historical feeding ecology: blood and feather δ13C and δ15N values document foraging plasticity of small sympatric petrels. Mar Ecol Prog Ser 505:267–280.

# Eizenberg YH, Fromant A, Lec'hvien A, Arnould JP (2021) Contrasting impacts of environmental variability on the breeding biology of two sympatric small procellariiform seabirds in south-eastern Australia. PLoS ONE 16(9):e0250916.

# Fromant A, Bost CA, Bustamante P, Carravieri A, Cherel Y, Delord K, et al. (2020) Temporal and spatial differences in the post-breeding behaviour of a ubiquitous Southern Hemisphere seabird, the common diving petrel. R Soc Open Sci 7(11):200670.

# Jouventin P, Mougin JL, Stahl JC, Weimerskirch H (1985) Comparative biology of the burrowing petrels of the Crozet Islands. Notornis 32:157–220.

# Lisovski S, Hewson CM, Klaassen RHG, Korner-Nievergelt F, Kristensen MW, Hahn S (2012) Geolocation by light: accuracy and precision affected by environmental factors. Methods Ecol Evol 3:603–612.

# Luque SP, Fried R (2011) Recursive filtering for zero offset correction of diving depth time series with gnu r package *divemove*. PLoS One 6(1):e15850.

# Phillips RA, Silk JRD, Croxall JP, Afanasyev V, Briggs DR (2004) Accuracy of geolocation estimates for flying seabirds. Mar Ecol Prog Ser 266:265–272.

# Qasem L, Cardew A, Wilson A, Griffiths I, Halsey LG, Shepard EL, et al. (2012) Tri-axial dynamic acceleration as a proxy for animal energy expenditure; should we be summing values or calculating the vector? PloS One 7(2):e31187.

# Marchant S, Higgins PJ (1990) Handbook of Australian, New Zealand and Antarctic birds. Melbourne, Australia: Oxford University Press.

# Payne MR, Prince PA (1979) Identification and breeding biology of the diving petrels *Pelecanoides georgicus* and *P. urinatrix exsul* at South Georgia. N Z J Zoo 6(2):299–318.

# Petalas C, Lazarus T, Lavoie RA, Elliott KH, Guigueno MF (2021) Foraging niche partitioning in sympatric seabird populations. Sci Rep 11:1–12.

# Post DM, Layman CA, Arrington DA, Takimoto G, Quattrochi J, Montana CG (2007) Getting to the fat of the matter: models, methods and assumptions for dealing with lipids in stable isotope analyses. Oecologia 152(1):179–189.

# R Development Core Team (2020) R: a language and environment for statistical computing, reference index version 2.12.2. See <http://www.R-project.org>.

# Sakamoto KQ, Sato K, Ishizuka M, Watanuki Y, Takahashi A, Daunt F, Wanless S (2009) Can ethograms be automatically generated using body acceleration data from free-ranging birds? PloS One 4(4):e5379.

# Spear LB, Ainley DG (1997) Flight behaviour of seabirds in relation to wind direction and wing morphology. Ibis 139:221–233.

# Wilson RP (2010) Resource partitioning and niche hyper‐volume overlap in free‐living pygoscelid penguins. Funct Ecol 24(3):646–657.

**Table S1: Summary of deployed tracking loggers and collected isotopic samples (whole blood and body feathers) from adult common and South Georgian diving petrels from Kerguelen Islands.** Abbreviation: SIA = stable isotope analysis.

| **Species** | **Status** | **Year** | **GPS/GLS** | |  | **Dive** | |  | **Accelerometry** | |  | **SIA** |
| --- | --- | --- | --- | --- | --- | --- | --- | --- | --- | --- | --- | --- |
|  |  |  | *Deployed*  *(retrieved)* | *Complete trips* |  | *Deployed*  *(retrieved)* | *Complete trips* |  | *Deployed*  *(retrieved)* | *Complete trips* |  |  |
| **Common diving petrels** | Incubation | 2015-2016 | 6 (4) | 0 |  |  |  |  |  |  |  | 10 |
|  |  | 2016-2017 |  |  |  |  |  |  |  |  |  | 12 |
|  |  | 2017-2018 |  |  |  |  |  |  |  |  |  |  |
|  |  | 2018-2019 | 8 (6) | 4 |  | 5 (5) | 5 |  | 5 (5) | 5 |  | 17 |
|  |  | 2019-2020 | 2 (2) | 2 |  | 5 (2) | 2 |  | 5 (2) | 2 |  | **7** |
|  |  | **Total** | **16 (12)** | **6** |  | **10 (7)** | **7** |  | **10 (7)** | **7** |  | **46** |
|  | Chick-rearing | 2015-2016 | 10 (10) | 6 |  |  |  |  |  |  |  | 10 |
|  |  | 2016-2017 |  |  |  |  |  |  |  |  |  |  |
|  |  | 2017-2018 | 15 (15) | 13 |  | 1 (1) | 2 |  |  |  |  | 15 |
|  |  | 2018-2019 | 8 (6) | 9 |  | 7 (7) | 13 |  | 7 (7) | 13 |  | 13 |
|  |  | 2019-2020 | 8 (8) | 11 |  | 4 (4) | 6 |  | 4 (4) | 6 |  | 12 |
|  |  | **Total** | **41 (39)** | **39** |  | **12 (12)** | **21** |  | **11 (11)** | **19** |  | **50** |
|  | Inter-breeding | 2015-2016 | 9 (6) | 0 |  |  |  |  |  |  |  | 20 |
|  |  | 2016-2017 |  |  |  |  |  |  |  |  |  | 12 |
|  |  | 2017-2018 | 12 (8) | **7** |  |  |  |  |  |  |  | 15 |
|  |  | 2018-2019 |  |  |  |  |  |  |  |  |  | 20 |
|  |  | 2019-2020 |  |  |  |  |  |  |  |  |  |  |
|  |  | **Total** | **21 (14)** | **7** |  |  |  |  |  |  |  | **67** |
| **South Georgian diving petrels** | Incubation | 2015-2016 |  |  |  |  |  |  |  |  |  | 7 |
|  |  | 2016-2017 |  |  |  |  |  |  |  |  |  |  |
|  |  | 2017-2018 |  |  |  |  |  |  |  |  |  |  |
|  |  | 2018-2019 | 10 (6) | 5 |  | 2 (0) | 0 |  | 2 (0) | 0 |  | 9 |
|  |  | 2019-2020 | 2 (1) | 1 |  | 4 (4) | 4 |  | 4 (4) | 4 |  | 6 |
|  |  | **Total** | **12 (7)** | **6** |  | **6 (4)** | **4** |  | **6 (4)** | **4** |  | **22** |
|  | Chick-rearing | 2015-2016 | 9 (9) | 9 |  |  |  |  |  |  |  | 7 |
|  |  | 2016-2017 |  |  |  |  |  |  |  |  |  |  |
|  |  | 2017-2018 | 19 (19) | 21 |  | 6 (5) | 5 |  |  |  |  | 23 |
|  |  | 2018-2019 | 9 (8) | 10 |  | 7 (6) | 8 |  | 7 (6) | 8 |  | 15 |
|  |  | 2019-2020 | 6 (6) | 6 |  |  |  |  |  |  |  | 6 |
|  |  | **Total** | **43 (42)** | **46** |  | **13 (11)** | **13** |  | **7 (6)** | **8** |  | **51** |
|  | Inter-breeding | 2015-2016 | 10 (3) | 0 |  |  |  |  |  |  |  | 20 |
|  |  | 2016-2017 |  |  |  |  |  |  |  |  |  | 12 |
|  |  | 2017-2018 | 8 (4) | 4 |  |  |  |  |  |  |  | 15 |
|  |  | 2018-2019 |  |  |  |  |  |  |  |  |  | 20 |
|  |  | 2019-2020 |  |  |  |  |  |  |  |  |  |  |
|  |  | **Total** | **18 (7)** | **4** |  |  |  |  |  |  |  | **67** |

**Table S2: Inter-sex comparison of morphological measurements, trip parameters and whole blood and body feather δ^13^C and δ^15^N values of common (CDP) and South Georgian (SGDP) diving petrels from Kerguelen Islands.** Values are means ± SD, and statistically significant results are highlighted in bold.

|  |  | **CDP**  **Female** | **CDP**  **Male** | **Test**  **CDP (Female vs Male)** | **SGDP**  **Female** | **SGDP**  **Male** | **Test**  **SGDP (Female vs Male)** |
| --- | --- | --- | --- | --- | --- | --- | --- |
| **Measurements** | **Body mass (g)** | 145 ± 8 (n = 16) | 146 ± 14 (n = 23) | *t*-test: *t_36.434_* = -0.367,  *P* = 0.716 | 131 ± 10 (n = 22) | 128 ± 11 (n = 23) | *t*-test: *t_42.939_* = 0.860,  *P* = 0.394 |
|  | **Wing length (mm)** | 127 ± 3 (n = 13) | 125 ± 5 (n = 18) | *t*-test: *t_28.757_* = 1.557,  *P* = 0.130 | 120 ± 3 (n = 20) | 118 ± 3 (n = 17) | *t*-test: *t_34.979_* = 2.304,  ***P* = 0.027** |
|  | **Tarsus length (mm)** | 26.3 ± 1.7 (n = 13) | 26.7 ± 1.3 (n = 18) | *t*-test: *t_22.537_* = -0.618,  *P* = 0.542 | 24.7 ± 0.9 (n = 20) | 24.8 ± 0.9 (n = 18) | *t*-test: *t_35.947_* = -0.143,  *P* = 0.887 |
|  | **Bill length (mm)** | 16.2 ± 0.4 (n = 13) | 16.2 ± 0.6 (n = 18) | *t*-test: *t_28.890_* = -0.408,  *P* = 0.687 | 15.4 ± 0.6 (n = 20) | 15.7 ± 0.7 (n = 18) | *t*-test: *t_34.071_* = -1.595,  *P* = 0.120 |
| **Incubation period** | **Trip duration (h)** | 37 ± 13 (n = 3) | 45 ± 2 (n = 3) | Mann-Whitney *U* test: *U* = 2, *P* = 0.800 | 60 ± 11 (n = 3) | 32 ± 21 (n = 3) | Mann-Whitney *U* test: *U* = 8, *P* = 0.200 |
|  | **Total distance travelled (km)** | 506 ± 400 (n = 3) | 506 ± 532 (n = 3) | Mann-Whitney *U* test: *U* = 2, *P* = 0.800 | 768 ± 101 (n = 3) | 452 ± 102 (n = 3) | Mann-Whitney *U* test: *U* = 9, *P* = 0.100 |
|  | **Maximum distance from colony (km)** | 217 ± 182 (n = 3) | 237 ± 198 (n = 3) | Mann-Whitney *U* test: *U* = 4, *P* = 1.000 | 379 ± 136 (n = 3) | 265 ± 32 (n = 3) | Mann-Whitney *U* test: *U* = 8, *P* = 0.200 |
|  | **Dive depth (m)** | 6.7 ± 0.5 (n = 5) | 6.2 ± 0.3 (n = 3) | Mann-Whitney *U* test: *U* = 12, *P* = 0.250 | 7.3 ± 0.4 (n = 2) | 6.0 ± 0.4 (n = 2) | na |
|  | **Dive duration (s)** | 29 ± 4 (n = 5) | 27 ± 1 (n = 3) | Mann-Whitney *U* test: *U* = 9, *P* = 0.786 | 27 ± 1 (n = 2) | 22 ± 4 (n = 2) | na |
|  | **Blood δ^13^C (‰)** | -22.0 ± 1.6 (n = 18) | -20.9 ± 2.2 (n = 26) | *t*-test: *t_41.847_* = -1.874,  *P* = 0.068 | -23.5 ± 0.2 (n = 10) | -23.2 ± 0.4 (n = 12) | *t*-test: *t_14.519_* = -1.625,  *P* = 0.126 |
|  | **Blood δ^15^N (‰)** | 8.6 ± 1.6 (n = 18) | 9.6 ± 1.3 (n = 26) | *t*-test: *t_41.378_* = -3.155,  ***P* = 0.003** | 9.0 ± 0.2 (n = 10) | 9.0 ± 0.3 (n = 12) | *t*-test: *t_14.929_* = 0.277,  *P* = 0.786 |
| **Chick-rearing period** | **Trip duration (h)** | 19 ± 1 (n = 13) | 18 ± 2 (n = 25) | Mann-Whitney *U* test: *U* = 195, *P* = 0.332 | 31 ± 12 (n = 17) | 24 ± 7 (n = 12) | Mann-Whitney *U* test: *U* = 132, *P* = 0.199 |
|  | **Total distance travelled (km)** | 84 ± 30 (n = 7) | 85 ± 23 (n = 14) | Mann-Whitney *U* test: *U* = 47, *P* = 0.913 | 579 ± 114 (n = 11) | 485 ± 99 (n = 10) | Mann-Whitney *U* test: *U* = 85, ***P* = 0.036** |
|  | **Maximum distance from colony (km)** | 18 ± 5 (n = 11) | 19 ± 6 (n = 19) | Mann-Whitney *U* test: *U* = 89, *P* = 0.524 | 210 ± 72 (n = 16) | 216 ± 69 (n = 19) | Mann-Whitney *U* test: *U* = 162, *P* = 0.756 |
|  | **Dive depth (m)** | 15.3 ± 2.8 (n = 6) | 15.5 ± 3.4 (n = 14) | Mann-Whitney *U* test: *U* = 40, *P* = 0.904 | 5.3 ± 1.0 (n = 6) | 7.2 ± 3.6 (n = 5) | Mann-Whitney *U* test: *U* = 10, *P* = 0.429 |
|  | **Dive duration (s)** | 44 ± 4 (n = 6) | 44 ± 6 (n = 14) | Mann-Whitney *U* test: *U* = 38, *P* = 0.779 | 22 ± 2 (n = 6) | 24 ± 8 (n = 5) | Mann-Whitney *U* test: *U* = 15, *P* = 1.000 |
|  | **Blood δ^13^C (‰)** | -18.8 ± 1.3 (n = 20) | -18.1 ± 1.1 (n = 25) | *t*-test: *t_37.315_* = -1.912,  *P* = 0.064 | -22.6 ± 0.4 (n = 23) | -22.7 ± 0.4 (n = 24) | *t*-test: *t_44.998_* = 0.751,  *P* = 0.457 |
|  | **Blood δ^15^N (‰)** | 10.9 ± 1.0 (n = 20) | 11.3 ± 0.7 (n = 25) | *t*-test: *t_34.128_* = -1.544,  *P* = 0.131 | 8.4 ± 0.3 (n = 23) | 8.4 ± 0.3 (n = 24) | *t*-test: *t_44.960_* = -0.021,  *P* = 0.983 |
| **Inter-breeding period** | **Post-breeding migration duration (days)** | 206 ± 19 (n = 4) | 210 ± 10 (n = 3) | Mann-Whitney *U* test: *U* = 7, *P* = 0.857 | 239 (n = 1) | 244 ± 6 (n = 3) | na |
|  | **Migration maximum range (km)** | 2 528 ± 256 (n = 6) | 2 341 ± 236 (n = 6) | Mann-Whitney *U* test: *U* = 27, *P* = 0.180 | 3 517 (n = 1) | 4 165 ± 326 (n = 4) | na |
|  | **Migration total distance travelled (km)** | 42 941 ± 6 248 (n = 4) | 45 954 ± 6 081 (n = 6) | Mann-Whitney *U* test: *U* = 4, *P* = 0.629 | 81 462 (n = 1) | 91 598 ± 9 975 (n = 3) | na |
|  | **Feathers δ^13^C (‰)** | -23.4 ± 1.9 (n = 28) | -23.2 ± 1.7 (n = 31) | *t*-test: *t_53.720_* = -0.387,  *P* = 0.700 | -21.6 ± 1.0 (n = 20) | -21.9 ± 0.6 (n = 25) | *t*-test: *t_29.477_* = 1.138,  *P* = 0.264 |
|  | **Feathers δ^15^N (‰)** | 8.5 ± 1.4 (n = 28) | 8.5 ± 1.0 (n = 31) | *t*-test: *t_48.846_* = -0.113,  *P* = 0.911 | 6.9 ± 1.8 (n = 20) | 6.6 ± 1.6 (n = 25) | *t*-test: *t_37.882_* = 0.562,  *P* = 0.578 |

**Table S3: Morphological measurements (mean ± SD) of adult common and South-Georgian diving petrels from Kerguelen Islands.** Individuals from both species were measured between 2016 and 2020.

|  | **Common diving petrels**  **(n = 39)** | **South Georgian diving petrels**  **(n = 43)** | **Test** |
| --- | --- | --- | --- |
| **Body mass (g)** | 147 ± 12 (122 – 186) | 128 ± 10 (110 – 150) | *t*-test: *t_65.916_* = 7.348, *P* < 0.001 |
| **Wing length (mm)** | 126 ± 4 (115 – 132) | 119 ± 3 (113 – 126) | *t*-test: *t_65.334_* = 8.383, *P* < 0.001 |
| **Tarsus length (mm)** | 26.4 ± 1.5 (22.5 – 29.1) | 24.6 ± 0.9 (23.0 – 27.1) | *t*-test: *t_59.883_* = 7.119, *P* < 0.001 |
| **Bill length (mm)** | 16.2 ± 0.5 (15.1 – 17.2) | 15.5 ± 0.8 (13.8 – 16.9) | *t*-test: *t_74.910_* = 5.096, *P* < 0.001 |

**Table S4: Whole blood and body feather δ^13^C and δ^15^N values (means ± SD) of common and South-Georgian diving petrels from Kerguelen Islands.** Significantly different values (Mann-Whitney *U* test: *P* < 0.05) are indicated by different superscript letters/symbols, for each year (row; difference between species-stage group; a, b, c or d), and for each species-stage group (columns; inter-annual variation; *, # or &). In order to allow statistical comparisons between blood and feathers, isotopic values of feathers were corrected using mean corrections factors from Cherel et al. (2014) (underlined values = corrected values).

|  |  | **Common diving petrels** | | |  | **South Georgian diving petrels** | | |
| --- | --- | --- | --- | --- | --- | --- | --- | --- |
|  |  | Inter-breeding  (feathers) | Incubation  (blood) | Chick-rearing  (blood) |  | Inter-breeding  (feathers) | Incubation  (blood) | Chick-rearing  (blood) |
| **δ^13^C**  **(‰)** | 2015-2016 | -23.1 ± 1.3^*^ (n = 20) | -22.2 ± 1.0^a*^ (n = 10) | -19.0 ± 1.2^b*^ (n = 10) |  | -21.3 ± 1.3^*^ (n = 12) | -23.2 ± 0.4^a*^ (n = 7) | -23.0 ± 0.5^a*^ (n = 7) |
|  | 2016-2017 | -23.5 ± 2.1^*^ (n = 12) | -20.6 ± 2.4^*#^ (n = 12) | - |  | - | - | - |
|  | 2017-2018 | -23.2 ± 1.4^*^ (n = 15) | - | -18.8 ± 1.0^a*^ (n = 15) |  | -21.8 ± 0.6^*^ (n = 23) | - | -22.4 ± 0.3^a#^ (n = 23) |
|  | 2018-2019 | -23.3 ± 2.2^*^ (n = 20) | -22.1 ± 1.7^a*^ (n = 17) | -17.8 ± 1.0^c*^ (n = 13) |  | -21.8 ± 0.8^*^ (n = 20) | -23.4 ± 0.3^b*^ (n = 9) | -22.9 ± 0.1^bd*^ (n = 15) |
|  | 2019-2020 | - | -19.2 ± 1.8^a#^ (n = 7) | -18.2 ± 1.5^a*^ (n = 12) |  | - | -23.1 ± 0.2^b*^ (n = 6) | -22.7± 0.2^b*#^ (n = 6) |
|  | **Means** | **-23.3 ± 1.7**  **-24.1 ± 1.7^a^** | **-21.3 ± 2.1^b^** | **-18.4 ± 1.2^c^** |  | **-21.7 ± 0.8**  **-22.5 ± 0.8^d^** | **-23.3 ± 0.3^e^** | **-22.7 ± 0.4^d^** |
|  |  |  |  |  |  |  |  |  |
|  | 2015-2016 | 8.8 ± 0.9^*^ (n = 20) | 9.3 ± 0.8^a*#^ (n = 10) | 11.2 ± 0.9^b*^ (n = 10) |  | 7.9 ± 2.0^*^ (n = 12) | 9.2 ± 0.2^a*^ (n = 7) | 8.8 ± 0.4^a*^ (n = 7) |
|  | 2016-2017 | 8.9 ± 1.2^*^ (n = 12) | 9.4 ± 1.5^*#^ (n = 12) | - |  | - | - | - |
| **δ^15^N**  **(‰)** | 2017-2018 | 8.3 ± 0.7^*^ (n = 15) | - | 10.8 ± 0.7^a*^ (n = 15) |  | 6.8 ± 1.5^#^ (n = 23) | - | 8.4 ± 0.2^b#^ (n = 23) |
|  | 2018-2019 | 8.3 ± 1.5^*^ (n = 20) | 8.7 ± 1.2^ab*^ (n = 17) | 11.4 ± 0.8^c*^ (n = 13) |  | 6.2 ± 1.3^#^ (n = 20) | 8.9 ± 0.2^b#^ (n = 9) | 8.4 ± 0.2^a#&^ (n = 15) |
|  | 2019-2020 | - | 10.1 ± 0.9^a#^ (n = 7) | 10.6 ± 1.4^a*^ (n = 12) |  | - | 8.7 ± 0.2^b#^ (n = 6) | 7.9 ± 0.4^c&^ (n = 6) |
|  | **Means** | **8.4 ± 1.1**  **7.5 ± 1.1^a^** | **9.2 ± 1.2^b^** | **11.0 ± 1.0^c^** |  | **6.5 ± 1.7**  **5.6 ± 1.7^d^** | **9.0 ± 0.3^b^** | **8.4 ± 0.4^e^** |
|  |  |  |  |  |  |  |  |  |


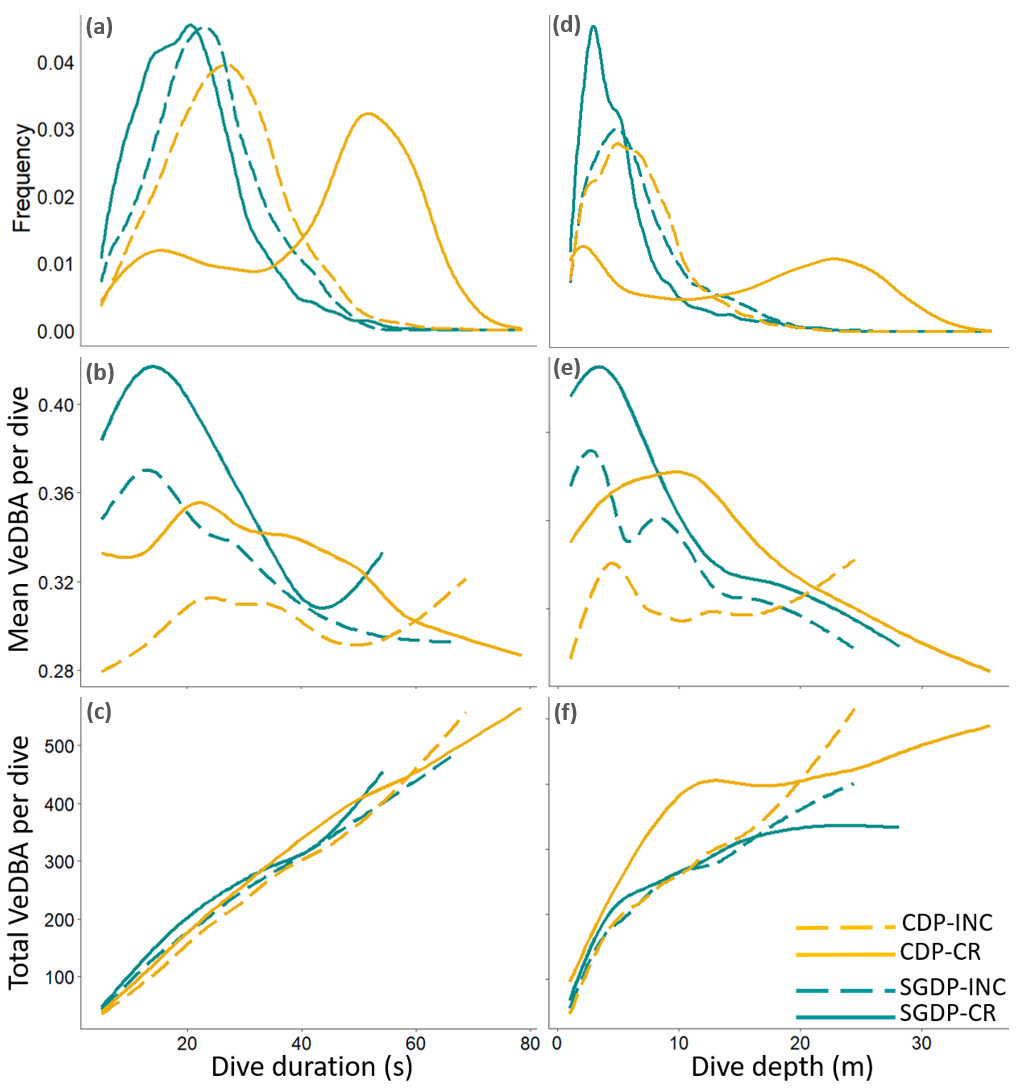


**Figure S1: Effect of dive duration (a, b, c) and dive depth (d, e, f) on mean and total VeDBA per dive predicted by generalized additive mixed models.** CDP-INC = common diving petrels during the incubation period; CDP-CR = common diving petrels during the chick-rearing period; SGDP-INC = South Georgian diving petrels during the incubation period; SGDP-CR = South-Georgian diving petrels during the chick-rearing period.


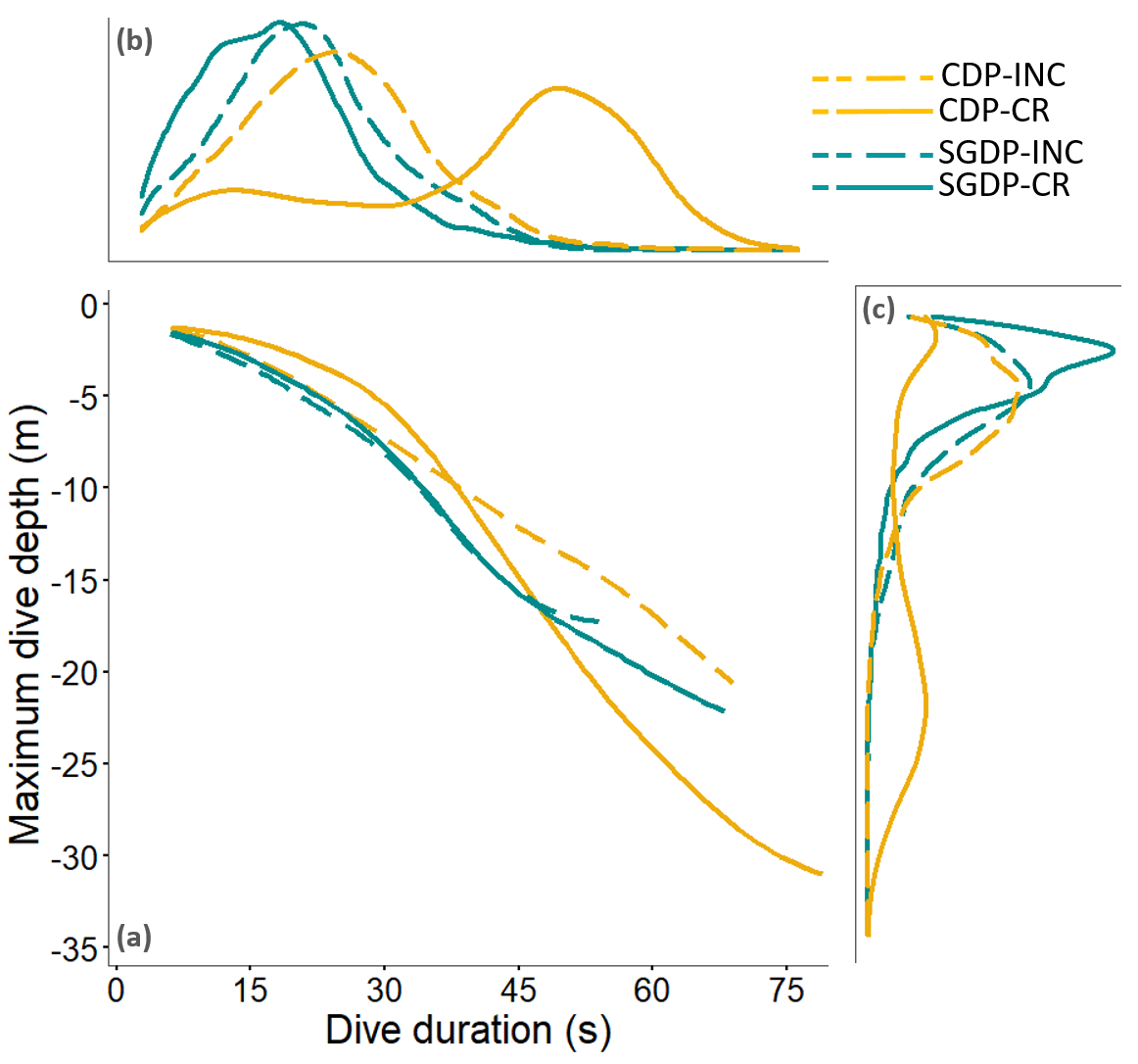


**Figure S2: Correlation between dive depth and dive duration (a) predicted by generalized additive mixed models.** Upper (b) and right (c) panels provide the data distribution of dive duration and dive depth, respectively. CDP-INC = common diving petrels during the incubation period; CDP-CR = common diving petrels during the chick-rearing period; SGDP-INC = South-Georgian diving petrels during the incubation period; SGDP-CR = South Georgian diving petrels during the chick-rearing period.

**
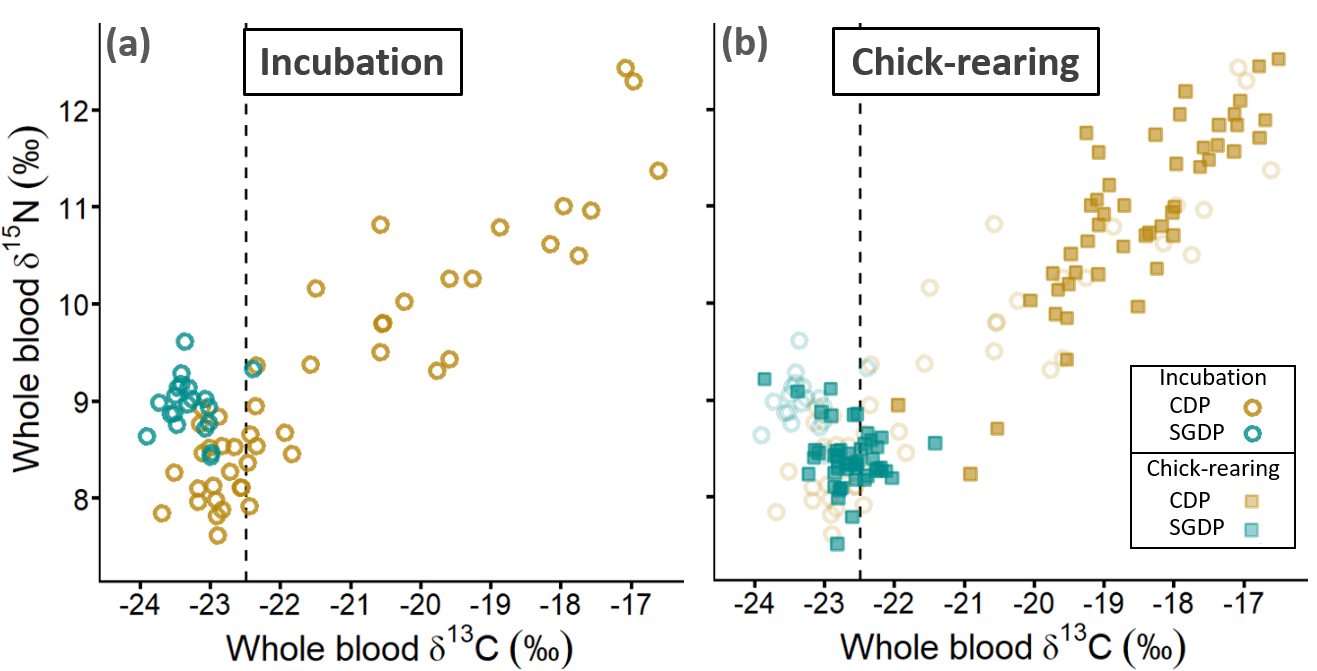
**

**Figure S3: Transition of the isotopic niches of common (CDP, yellow) and South-Georgian (SGDP, blue) diving petrels from the incubation (a) to the chick-rearing period (b).** Panel (a) shows δ^13^C and δ^15^N values in blood during the incubation period only (open circle); and panel (b) corresponds to isotopic values during the chick-rearing period (full squares), with values during the incubation period in back-ground (faded open circle). The black vertical dashed line corresponds to the value for the Polar Front.
